# Fine needle aspiration biopsy diagnosis of metastatic neoplasms of the breast. A three-case report

**DOI:** 10.1186/1742-6413-2-17

**Published:** 2005-09-20

**Authors:** Garza-Guajardo Raquel, Mendez-Olvera Nora, Flores-Gutierrez Juan Pablo, Hernandez-Martinez Silvia, Candanosa-Mc Cann Michelle, Ancer-Rodriguez Jesús, Barboza-Quintana Oralia

**Affiliations:** 1 Anatomical Pathology and Cytopathology department, University Hospital "Dr. José E.Gonzalez", UANL, Monterrey, Mexico

## Abstract

Metastases to the breast are unusual lesions that make up approximately 2% of all malignant mammary neoplasms and may mimic both benign and malignant primary neoplasms from a clinical point of view, as well as in imaging studies. Arriving at a correct diagnosis is therefore essential in order to establish appropriate management.

We present three cases of metastatic neoplasms diagnosed through fine needle aspiration biopsy and immunocytochemistry. The cytological diagnoses were: medulloblastoma in an 18-year-old woman, melanoma in a 26-year-old man, and an exceptional case of ovarian sarcoma originating from a granulosa cell tumor with metastases to both breasts. A metastatic disease should be considered in the differential diagnosis of a palpable mass in the breast, especially if there is a history of an extramammary malignant neoplasm. Fine needle aspiration biopsy is the method of choice for the management of these cases.

Whenever possible the exam of the material obtained should be compared to the previous biopsy, which is usually enough to arrive at a correct diagnosis, thus preventing unnecessary surgical procedures.

## Introduction

Metastatic neoplasms of the breast are very rare [1,2]. They may mimic benign and malignant primary neoplasms both clinically and radiologically [3], and the clinical diagnosis often goes unsuspected, especially when the first manifestation of the clinical picture is a breast tumor and the existence of a primary neoplasm is unknown or not taken into consideration [4]. In most cases, management through fine needle aspiration biopsy (FNAB) permits the correct diagnosis of a metastatic neoplasm, which prevents unnecessary radical surgeries and allows us to start appropriate systemic treatment promptly [5].

For FNAB evaluation it is necessary to know the patient's clinical history. It will usually be easier to achieve a definitive diagnosis when the history of extramammary malignant neoplasm is known. Therefore, metastatic lesions should be considered in the differential diagnosis of tumors of the breast, particularly in those cases presenting unusual clinical, radiological, or cytological characteristics. For the latter, it is very useful to perform immunocytochemistry stains on the smears or cell blocks to arrive at a definitive diagnosis.

## Materials and methods

Three cases of metastatic neoplasms of the breast were identified through FNAB, from a total 639 aspirates of breast tumors performed between January 2001 and December 2003 at the Aspiration Room of the Anatomical Pathology and Cytopathology Service of the Hospital Universitario. The pathologist performed all the procedures by using a gauge 22 needle in a 10 ml syringe. One or two punctures were performed in each case, with an average of four smears per patient. An air-dried smear was stained with Diff-Quik for immediate evaluation of the sample, and the rest were fixed with 95° alcohol and stained by Papanicolau's method. Additionally, immunohistochemistry stains were performed on the cytological material [Table 1].

**Table 1:** Immunochemical markers used:

Antibody	Company	Dilution	HIER
CD 45	DAKO	1:100	yes
ENE	DAKO	1:200	no
S-100 Protein	DAKO	1:200	yes
HMB 45	DAKO	1:50	yes
Vimentin	DAKO	1:200	yes
Cytokeratin AE1	DAKO	1:200	yes
CA 15-3	DAKO	1:250	yes

HIER: Heat induce epitope retrieval

## Results

Out of a total 639 aspiration biopsies of the breast, 3 cases (0.4%) were diagnosed as metastatic neoplasms. In the three cases, immunochemistry was performed on the smears to corroborate the diagnosis, and the histological slides of the primary tumor were reviewed in order to compare them with the original morphology.

### Case description

#### Case 1

An 18-year-old female patient with a history of cerebellar medulloblastoma diagnosed 18 months before admission. At the time she was treated with subtotal surgical resection and the placement of a ventricular peritoneal shunt, in addition to radiotherapy. This time she presented with a tumor of the right breast and bone pain.

The physical examination revealed a lobulated tumor of the breast, approximately 3 cm in diameter, with poorly defined margins, no changes in the overlying skin and no axillary or supraclavicular adenopathies. An FNAB procedure was performed on the tumor, which yielded abundant material consisting of a population of non-cohesive, small, round cells with scant cytoplasm and a high nucleus-cytoplasm ratio. The nuclei were rounded and some of them were elongated, with lumpy chromatin and small nucleoli.

**Figure 1:**
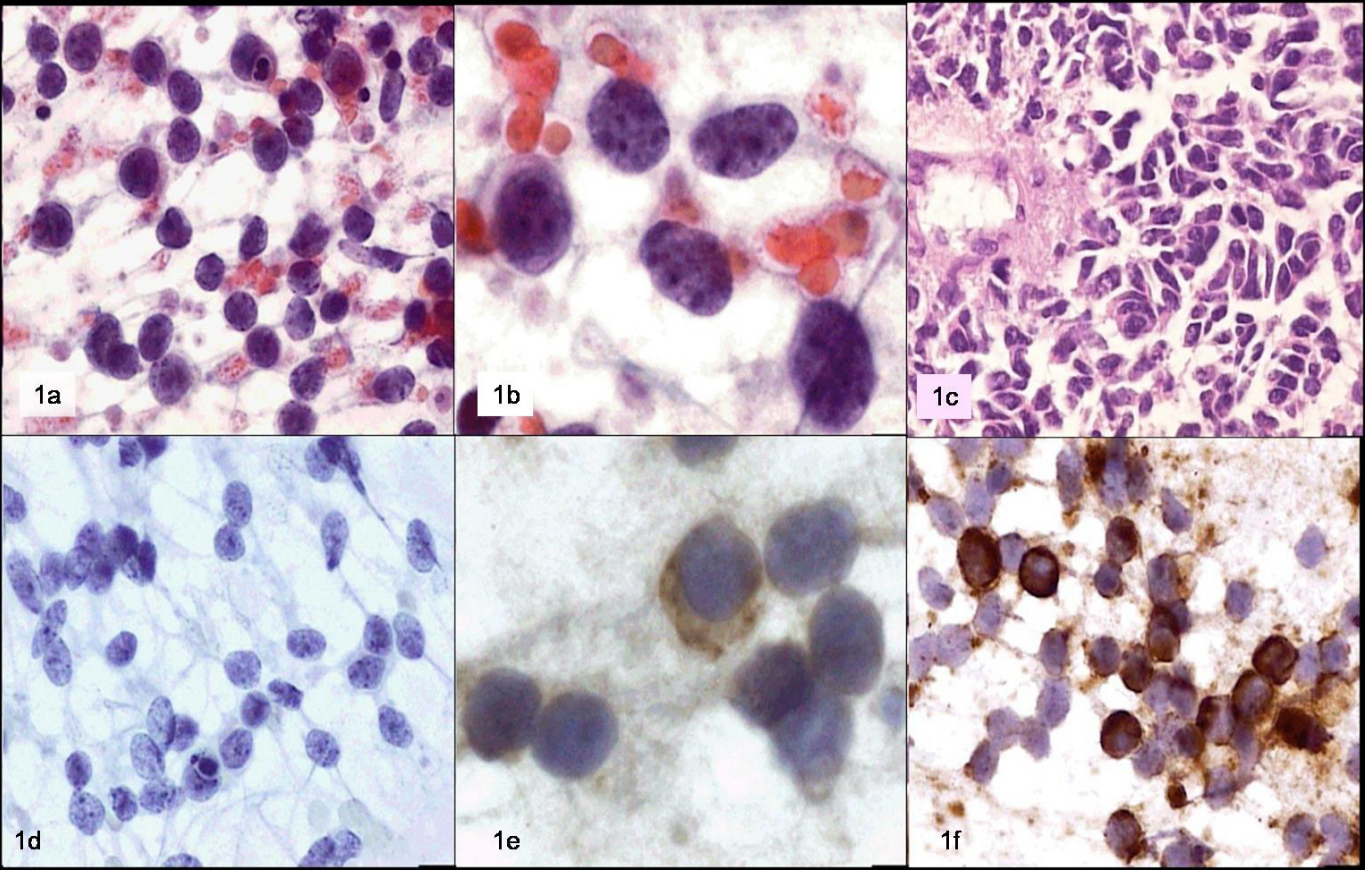
a y b: Papamicolaou; c: biopsy; d: CD45; e: neuron specific enolase; f: vimentin.

Immunocytochemistry was performed, which was positive for neuron-specific enolase and negative for the common leukocyte antigen. The cytological diagnosis was metastatic medulloblastoma. Fig. 1

### Case 2

A 26-year-old man with a history of being diagnosed with a skin melanoma three years earlier, he presents with a firm, poorly-defined, multinodular retroareolar mass, approximately 5 cm in diameter. The smears showed numerous cells, predominantly epithelioid-looking, forming slightly cohesive groups, as well as isolated polygonal cells with cytoplasmic elongations and nuclei with acidophilic macronucleoli and pseudoinclusions. Scant cells with melanotic pigment were identified.

**Figure 2:**
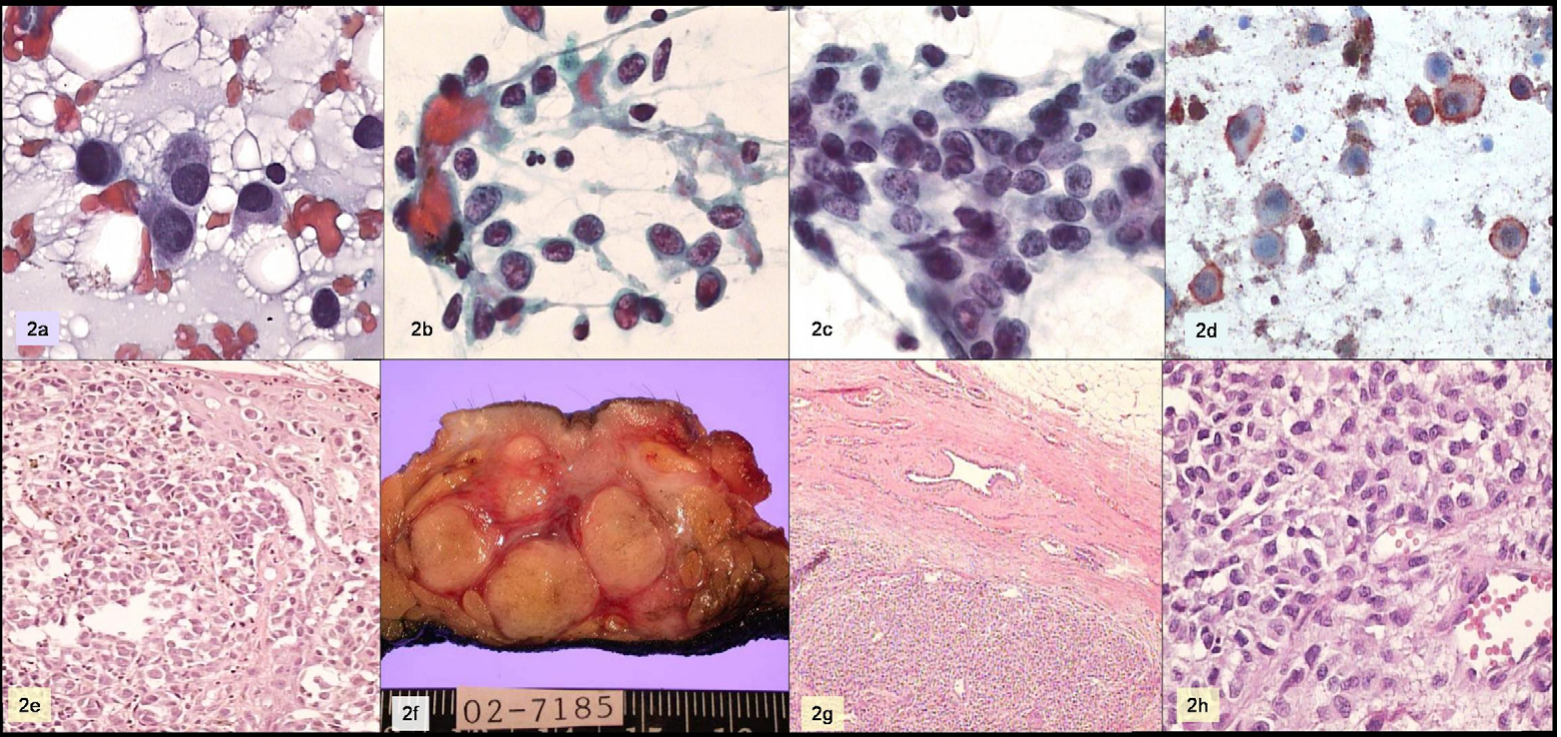
a, b, c: Papanicolaou; d: HMB-45; e: skin biopsy; f, g, h: breast tumor.

Immunohistochemistry was performed, with the following results: negative for cytokeratin, positive for vimentin, S-100 protein, and HMB45. A metastatic melanoma was diagnosed. Fig. 2

#### Case 3

A 55-year-old woman who was diagnosed with a granulosa cell tumor of the ovary with sarcomatous differentiation a year prior to her admission, at present she shows several fastgrowing nodular tumors in both breasts, as well as imaging suggesting metastasis to the liver. FNAB of both breasts was performed.

**Figure 3:**
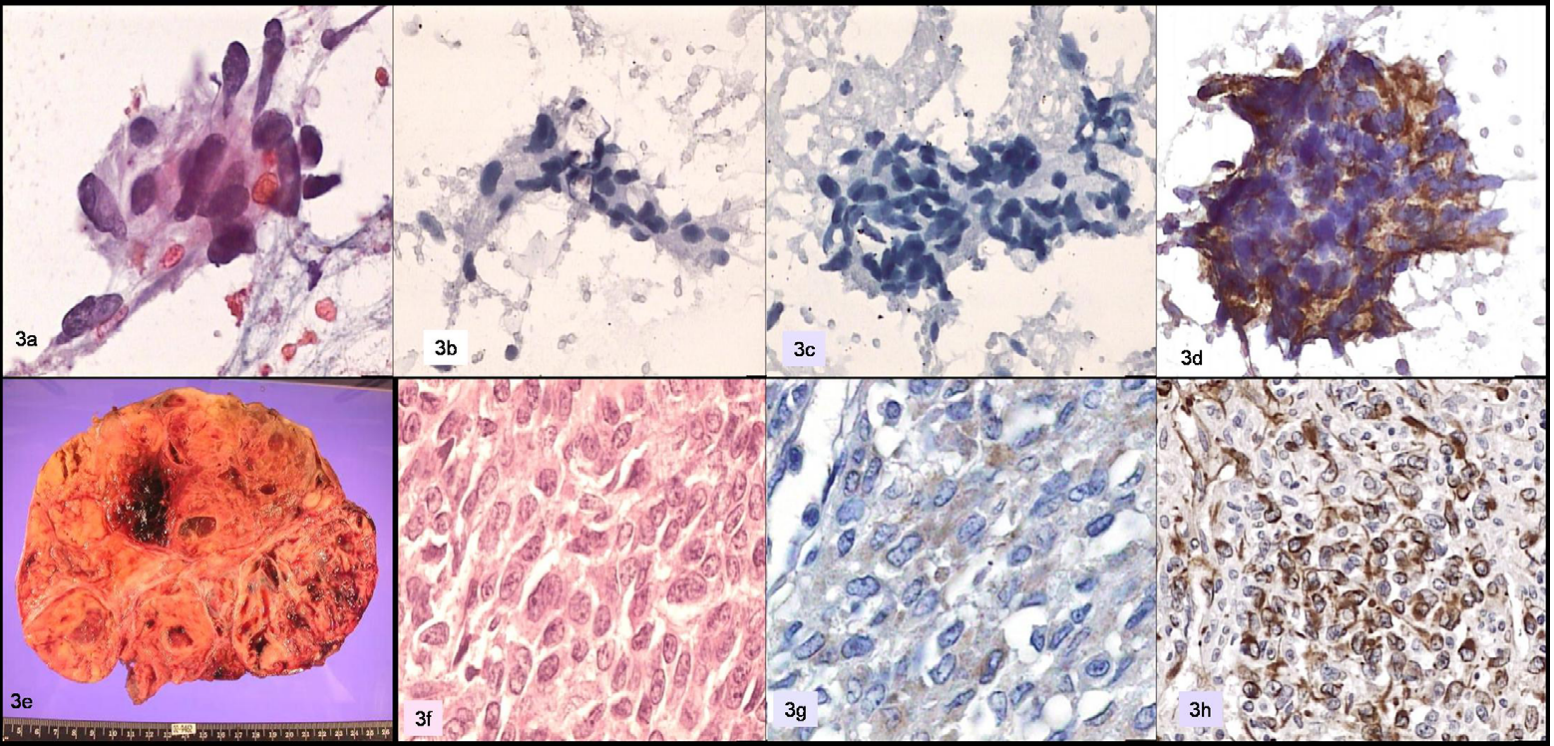
a: Papanicolaou; b: cytokeratin; c: CA 15-3; d: vimentin; e, f: ovarian tumor; g: inhibin; h: WT1.

The smears showed numerous neoplastic cells, predominantly spindle-shaped cells forming small groups, intermingled with other pleomorphic and isolated polygonal cells with large nuclei and no nucleoli. Immunohistochemistry was negative for cytokeratin and CA 15-3, and positive for vimentin. A metastatic sarcoma was diagnosed. Fig 3.

## Discussion

Metastases to the breast of neoplasms originating in organs other than the breast are very rare. In a series of 2529 breast aspiration biopsies, 666 were diagnosed as malignant neoplasms, and only 18 (2.7%) were of extramammary origin [5]. The identification of a secondary neoplasm in the breast is clinically complicated because secondary neoplasms appear as well-circumscribed rounded masses, most often located in the upper outer quadrant, and without the spiculations or microcalcifications of primary carcinomas [6]. On the other hand, the presence of well-circumscribed multiple or bilateral nodules may suggest a benign process in the mammography [7].

Since metastatic neoplasms may mimic primary tumors, it is important to know the patient's clinical history when interpreting the FNAB. However, for patients with unknown primary neoplasms the metastatic nature of the lesion should be suspected when we find unusual cytological patterns. Immunocytochemistry techniques are undoubtedly of great use in the latter cases.

Some clinical characteristics help us identify neoplasms of the breast that have a metastatic origin: the time span for the development of metastasis in patients with previously treated cancers is approximately two years. Metastases are usually to other sites or are detected simultaneously; by contrast, isolated metastases limited to the breast are infrequent [8].

Although virtually any malignant neoplasm can metastasize to the breast, this is most common for melanomas, carcinomas of the lung, neuroendrocrine and carcinoid carcinomas, ovarian carcinomas, and lymphoid and hematopoietic neoplasms [5,8-11]. Extracranial metastases of medulloblastoma occur in 5% to 7% of cases, and they are mainly related to ventricular peritoneal shunts. Most frequently affected sites are the bones, the lymphatic nodes, the liver, and the lungs, with isolated cases reported for other organs, such as the breast, for which only five cases have been described in the literature. Three of these cases were diagnosed through fine needle aspiration biopsy [12-16].

The cytological differential diagnosis includes lobular carcinoma, small-cell carcinoma, carcinoid tumor, lymphoma, and leukemic infiltration. Melanomas are some of the neoplasms that most often metastasize to the breast. Such metastases may be hard to recognize if the primary tumor is hiding or if there is no history of this tumor [17,18].

In addition, there is only one reported case of granulosa cell ovarian tumor metastatic to the breast diagnosed through a biopsy [19], and there are no previous reports of cases diagnosed with FNAB.

## Conclusion

Metastatic disease should be taken into consideration in the differential diagnosis of a tumor of the breast, especially if there is a history of an extramammary malignant neoplasm. FNAB, in combination with Immunohistochemistry, is the method of choice for the initial management of these cases. On the other hand, comparing the cytological findings with the previous biopsy will be usually enough to arrive at a correct diagnosis, thus preventing unnecessary radical surgical procedures and allowing us to establish the treatment of choice promptly and safely.
